# Supplementary material for: Human olfactory-auditory integration requires phase synchrony between sensory cortices
Source: Nat Commun. 2019 Mar 11;10:1168. doi: 10.1038/s41467-019-09091-3 (PMC6411726; doi:10.1038/s41467-019-09091-3)
Supplement: Supplementary file 1 — Supplementary Information [file 41467_2019_9091_MOESM1_ESM.pdf]

Supplementary Information for

Human olfactory-auditory integration requires phase synchrony between sensory cortices

Guangyu Zhou, Gregory Lane, Torben Noto, Ghazaleh Arabkheradmand, Jay A. Gottfried, Stephan U. Schuele, Joshua M. Rosenow, Jonas K. Olofsson, Donald A. Wilson, Christina Zelano.

Correspondence to: guangyu.zhou@northwestern.edu

This PDF file includes:

Supplementary Figure 1

Supplementary Figure 2

Supplementary Figure 1.

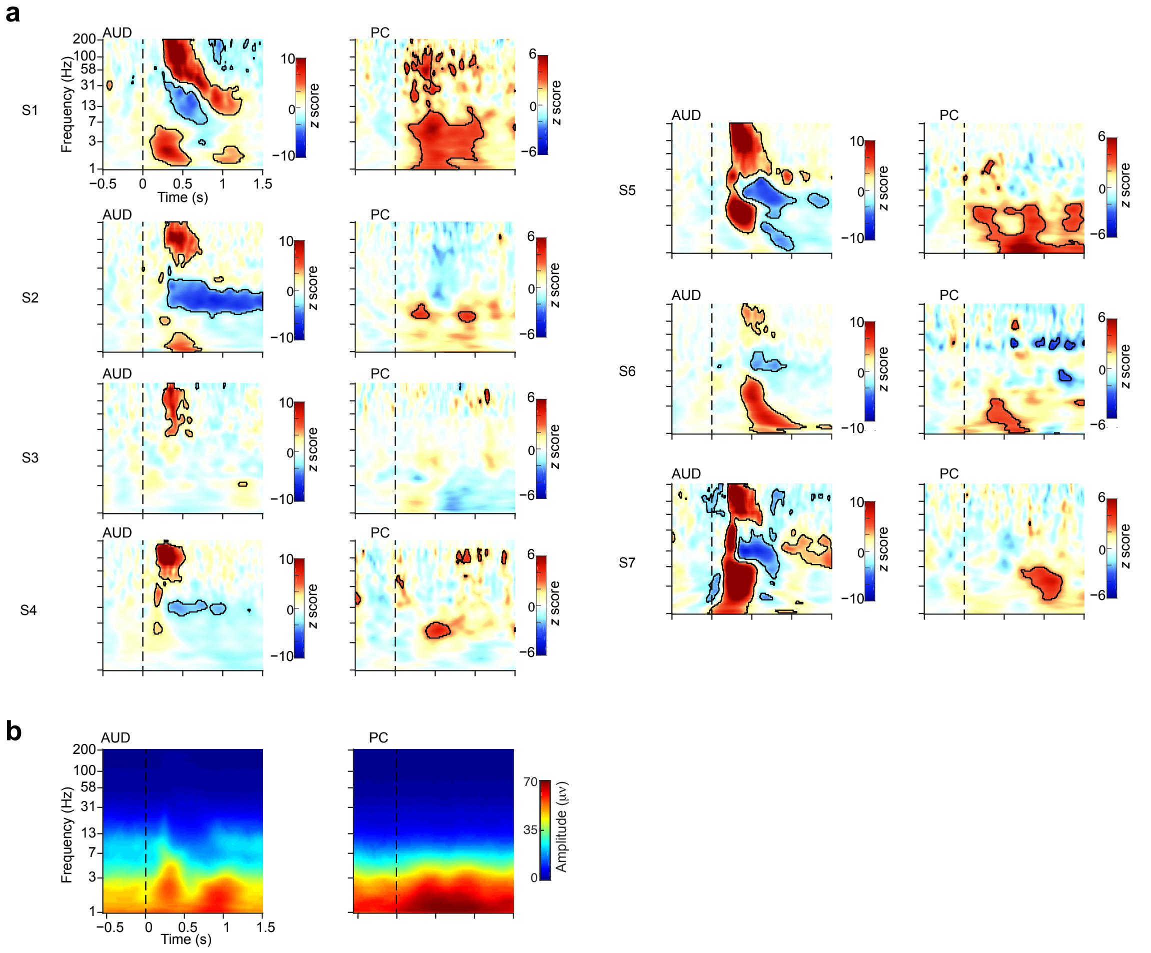

Supplementary Figure 1. Individual time-frequency analysis results and group-level spectrogram with no baseline correction. Dashed lines indicate auditory cue onset in all panels. (**a**) Spectrograms indicate *z* score maps of auditory cue-induced local field potential amplitude changes in auditory cortex (AUD) and piriform cortex (PC) for each participant (S1–S7). Statistically significant clusters outlined in black (FDR corrected $p < 0.05$, permutation test). (**b**) Group-level spectrogram without baseline correction (related to Figure 2a). Amplitude time series were obtained from the concatenated time series of all participants, segmented into epochs, and averaged over trials.

Supplementary Figure 2.

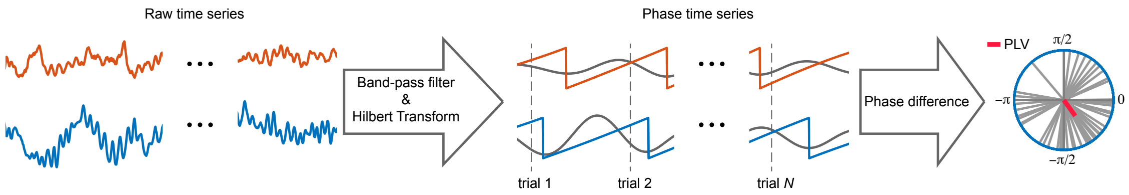

Supplementary Figure 2. Phase locking value (PLV) analysis schematic. Raw time series (left) were band-pass filtered at a specific frequency range for each channel (blue and red) separately. Then, instantaneous phase time-series were obtained from filtered data (solid gray lines) using the Hilbert Transform method (middle). The phase difference between the two channels was calculated for all trials (trial 1...*N*) at each time point. Finally, PLV was calculated as the average over all trials (right). PLV measures the consistency of this phase difference across trials and its value ranges from 0 (no consistency) to 1 (identical phase difference).
