# Supplementary material for: Human olfactory-auditory integration requires phase synchrony between sensory cortices
Source: Nat Commun. 2019 Mar 11;10:1168. doi: 10.1038/s41467-019-09091-3 (PMC6411726; doi:10.1038/s41467-019-09091-3)
Supplement: Supplementary file 3 — Reporting Summary [file 41467_2019_9091_MOESM3_ESM.pdf]

nature research

Corresponding author(s): Guangyu Zhou, Christina Zelano

Reporting Summary

Nature Research wishes to improve the reproducibility of the work that we publish. This form provides structure for consistency and transparency in reporting. For further information on Nature Research policies, see Authors & Referees and the Editorial Policy Checklist.

Statistical parameters

When statistical analyses are reported, confirm that the following items are present in the relevant location (e.g. figure legend, table legend, main text, or Methods section).

n/a	Confirmed	
☐	☒	The exact sample size (n) for each experimental group/condition, given as a discrete number and unit of measurement
☐	☒	An indication of whether measurements were taken from distinct samples or whether the same sample was measured repeatedly
☐	☒	The statistical test(s) used AND whether they are one- or two-sided<br>*Only common tests should be described solely by name; describe more complex techniques in the Methods section.*
☒	☐	A description of all covariates tested
☐	☒	A description of any assumptions or corrections, such as tests of normality and adjustment for multiple comparisons
☐	☒	A full description of the statistics including central tendency (e.g. means) or other basic estimates (e.g. regression coefficient) AND variation (e.g. standard deviation) or associated estimates of uncertainty (e.g. confidence intervals)
☐	☒	For null hypothesis testing, the test statistic (e.g. F, t, r) with confidence intervals, effect sizes, degrees of freedom and P value noted<br>*Give P values as exact values whenever suitable.*
☒	☐	For Bayesian analysis, information on the choice of priors and Markov chain Monte Carlo settings
☒	☐	For hierarchical and complex designs, identification of the appropriate level for tests and full reporting of outcomes
☐	☒	Estimates of effect sizes (e.g. Cohen's d, Pearson's r), indicating how they were calculated
☐	☒	Clearly defined error bars<br>*State explicitly what error bars represent (e.g. SD, SE, CI)*

Our web collection on statistics for biologists may be useful.

Software and code

Policy information about availability of computer code

Data collection	Matlab (R2016b); Clinical Nihon Kohden system.
Data analysis	Custom written Matlab code. Software used includes Matlab (R2016b); fieldtrip (20180429); SPM12; FSL (5.0.10); Freesurfer (v6.0.0).

For manuscripts utilizing custom algorithms or software that are central to the research but not yet described in published literature, software must be made available to editors/reviewers upon request. We strongly encourage code deposition in a community repository (e.g. GitHub). See the Nature Research guidelines for submitting code & software for further information.

Data

Policy information about availability of data

All manuscripts must include a data availability statement. This statement should provide the following information, where applicable:

- Accession codes, unique identifiers, or web links for publicly available datasets
- A list of figures that have associated raw data
- A description of any restrictions on data availability

All raw data and code will be made available upon reasonable request.

Field-specific reporting

Please select the best fit for your research. If you are not sure, read the appropriate sections before making your selection.

☒ Life sciences ☐ Behavioural & social sciences ☐ Ecological, evolutionary & environmental sciences

For a reference copy of the document with all sections, see nature.com/authors/policies/ReportingSummary-flat.pdf

Life sciences study design

All studies must disclose on these points even when the disclosure is negative.

Sample size	Sample sizes for iEEG studies depend on variability in clinically-determined electrode coverage areas, regions of interest, and stringency in statistical analyses, including requiring findings to be present consistently in each individual participant. Standard clinical electrode coverage at Northwestern Memorial Hospital includes medial limbic areas, including anterior amygdala and piriform cortex. Additionally, standard electrode coverage often includes a grid or depth wires reaching auditory cortex. In order to aggregate data within a given brain region, we require a minimum of 3 participants with electrodes implanted in that brain region. However, we held our analyses to the most stringent standards by conducting statistical tests at the individual level, in each participant separately. This required findings to be present in each individual, and consistent across all individuals. We found that a minimum of 5 participants provided sufficient power to conduct statistics across subjects. Based on our previous work and recent studies using iEEG techniques published in comparable journals, typical sample sizes for these types of data sets range from 3 participants to 10 participants.
Data exclusions	No participant's data was excluded. Trials and channels with excessive noise were excluded for each participant individually based on automated and manual inspection.
Replication	The main finding of auditory cue induced low-frequency amplitude change and phase synchrony between piriform cortex and auditory cortex was observed at the individual level, and statistical analyses were conducted both at the group level and at the individual level, in each participant. The reported findings were consistent across individuals. But we didn't replicate our findings in a different cohort of participants.
Randomization	No randomization was needed because each participant completed only one experimental task.
Blinding	Not relevant in this study.

Reporting for specific materials, systems and methods

Materials & experimental systems

n/a	Involved in the study
☒	☐ Unique biological materials
☒	☐ Antibodies
☒	☐ Eukaryotic cell lines
☒	☐ Palaeontology
☒	☐ Animals and other organisms
☐	☒ Human research participants

Methods

n/a	Involved in the study
☒	☐ ChIP-seq
☒	☐ Flow cytometry
☒	☐ MRI-based neuroimaging

Human research participants

Policy information about studies involving human research participants

Population characteristics	Seven (4 women) epilepsy patients were included in this study. Their ages at surgery ranged from 25 to 36 years (average: 33 years). The average duration of epilepsy was 9.86 years (ranging from 2–36 years).
Recruitment	In-patients at Northwestern Memorial Hospital Epilepsy Monitoring Unit were recruited based on patients' consent to participant in the study.
